# Preleukemic and second-hit mutational events in an acute myeloid leukemia patient with a novel germline *RUNX1* mutation

**DOI:** 10.1186/s40364-018-0130-2

**Published:** 2018-05-11

**Authors:** Isaac KS Ng, Joanne Lee, Christopher Ng, Bustamin Kosmo, Lily Chiu, Elaine Seah, Michelle Meng Huang Mok, Karen Tan, Motomi Osato, Wee-Joo Chng, Benedict Yan, Lip Kun Tan

**Affiliations:** 1 Yong Loo Lin School of Medicine, National University of Singapore, 1E Kent Ridge Road, Singapore, 119228 Singapore; 2 Department of Haematology-Oncology, National University Cancer Institute, National University Health System, 1E Kent Ridge Road, NUHS Tower Block, Level 7, Singapore, 119228 Singapore; 3 Molecular Diagnosis Centre, Department of Laboratory Medicine, National University Health System, 5 Lower Kent Ridge Road, Singapore, 119074 Singapore; 4 Cancer Science Institute of Singapore, National University of Singapore, 14 Medical Drive, Singapore, 117599 Singapore; 5 International Research Center for Medical Sciences, Kumamoto University, 2-2-1 Honjo, Chuo-ku, Kumamoto City, 860-0811 Japan; 6 Institute of Bioengineering and Nanotechnology, A*STAR, 31 Biopolis Way, Singapore, 138669 Singapore; 7 Department of Paediatrics, Yong Loo Lin School of Medicine, National University of Singapore, 1E Kent Ridge Road, NUHS Tower Block, Level 12, Singapore, 119228 Singapore; 8 Department of Medicine, Yong Loo Lin School of Medicine, National University of Singapore, 1E, Kent Ridge Road, NUHS Tower Block Level 10, Singapore, 119228 Singapore

**Keywords:** Familial platelet disorder, Acute myeloid Leukaemia, RUNX1, Stem cell transplant

## Abstract

**Background:**

Germline mutations in the *RUNX1* transcription factor give rise to a rare autosomal dominant genetic condition classified under the entity: Familial Platelet Disorders with predisposition to Acute Myeloid Leukaemia (FPD/AML). While several studies have identified a myriad of germline RUNX1 mutations implicated in this disorder, second-hit mutational events are necessary for patients with hereditary thrombocytopenia to develop full-blown AML. The molecular picture behind this process remains unclear. We describe a patient of Malay descent with an unreported 7-bp germline *RUNX1* frameshift deletion, who developed second-hit mutations that could have brought about the leukaemic transformation from a pre-leukaemic state. These mutations were charted through the course of the treatment and stem cell transplant, showing a clear correlation between her clinical presentation and the mutations present.

**Case presentation:**

The patient was a 27-year-old Malay woman who presented with AML on the background of hereditary thrombocytopenia affecting her father and 3 brothers. Initial molecular testing revealed the same novel *RUNX1* mutation in all 5 individuals. The patient received standard induction, consolidation chemotherapy, and a haploidentical stem cell transplant from her mother with normal *RUNX1* profile. Comprehensive genomic analyses were performed at diagnosis, post-chemotherapy and post-transplant. A total of 8 mutations (*RUNX1*, *GATA2*, *DNMT3A*, *BCORL1*, *BCOR*, 2 *PHF6* and *CDKN2A*) were identified in the pre-induction sample, of which 5 remained (*RUNX1*, *DNMT3A*, *BCORL1*, *BCOR* and 1 out of 2 *PHF6*) in the post-treatment sample and none were present post-transplant. In brief, the 3 mutations which were lost along with the leukemic cells at complete morphological remission were most likely acquired leukemic driver mutations that were responsible for the AML transformation from a pre-leukemic germline *RUNX1*-mutated state. On the contrary, the 5 mutations that persisted post-treatment, including the germline *RUNX1* mutation, were likely to be part of the preleukemic clone.

**Conclusion:**

Further studies are necessary to assess the prevalence of these preleukemic and secondary mutations in the larger FPD/AML patient cohort and establish their prognostic significance. Given the molecular heterogeneity of FPD/AML and other AML subtypes, a better understanding of mutational classes and their involvement in AML pathogenesis can improve risk stratification of patients for more effective and targeted therapy.

## Background

Acute myeloid leukemia (AML) is a molecularly heterogeneous clonal disease that requires the accumulation of at least two classes of gene mutations in its development [1, 2]. Currently, mutations identified in AML can be stratified into the conventional Class I (*FLT3*, *KRAS*, *KIT*) and Class II (*NPM1*, *CEBPA*, *RUNX1)* mutations that affect cell signaling genes and transcription factors respectively [1–3], as well as emerging groups of mutations involving epigenetic modifiers (*ASXL1*, *DNMT3A*, *EZH2*, *IDH1/2*, *TET2*), tumor suppressors (*TP53*, *WT1*) and spliceosomes (*SRSF2, ZRSR2*) [4, 5].

Germline mutations in the *RUNX1* transcription factor have a distinct causal implication in a rare entity of myeloid neoplasms known as Familial Platelet Disorder with predisposition to Acute Myeloid Leukemia (FPD/AML) [6–10]. This is an autosomal dominant genetic condition typically characterized by hereditary thrombocytopenia and qualitative platelet dysfunction that is associated with a high rate (approximately 40%) of AML transformation [6, 11]. While several previous studies have identified a myriad of germline *RUNX1* mutations implicated in FPD/AML (Table 1), second-hit mutational events are necessary for patients with hereditary thrombocytopenia to develop full-blown AML. However, the molecular picture behind this transformation process remains unclear. We herein describe an FPD/AML patient with a hitherto unreported 7-bp germline *RUNX1* frameshift deletion (NM_001754.4:c.554_560delAAGTCGC; NP_001745.2:p.Gln185ProfsTer24) who was diagnosed and treated at our hematological practice. We also sought to identify second-hit mutational events that could have brought about the AML transformation from a pre-leukemic germline *RUNX1*-mutated state.

**Table 1:** Studies reporting germline *RUNX1* mutations in FPD/AML

Year	Reference	Key Findings
1999	Song et al. [6]	Detection of heterozygous RUNX1 mutations in six FPD/AML pedigrees, mostly found within the Runt domain.
2001	Buijs et al. [10]	A novel missense mutation identified in RUNX1 Runt domain in FPD/AML.
2002	Walker et al. [8]	A novel heterozygous point mutation (A107P) identified in RUNX1 Runt domain in FPD/AML.
2002	Michaud et al. [7]	Three new heterozygous RUNX1 point mutations reported in FPD/AML, including the first familial RUNX1 mutation outside the Runt domain, in the C-terminal.
2005	Heller et al. [39]	A novel RUNX1 C-terminal point mutation identified in one FPD/AML pedigree.
2008	Beri-Dexheimer et al. [15]	Two novel germline RUNX1 mutations reported, including a heterozygous 8-bp deletion (c.442_449del) identified in FPD/AML.
2008	Owen et al. [40]	5 new cases of germline RUNX1 mutations reported in FPD/AML, including 3 N-terminal and 2 C-terminal mutations.
2009	Preudhomme et al. [24]	Detection of 4 germline RUNX1 mutations in 4 FPD/AML families, mostly within the Runt domain. Most importantly, this is the first report of second-hit RUNX1 somatic mutations identified in germline RUNX1-mutant AML.

## Case presentation

**Fig. 1:**
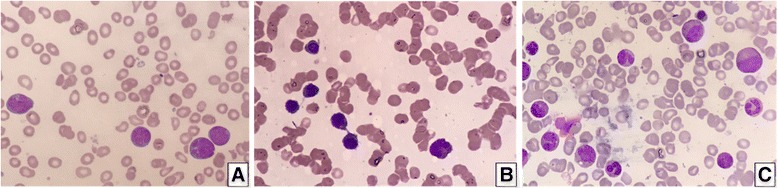
**a** Bone marrow aspirate at diagnosis depicting red cell anisopoikilocytosis, hypogranular platelets, myeloblasts and monoblasts/promonocytes. **b** Bone marrow aspirate after induction chemotherapy depicting red cell dysplasia with intercytoplasmic bridging and occasional myeloblasts. **c** Bone marrow aspirate after stem cell transplant depicting a normal haematopoietic maturation and no myeloblasts or monoblasts seen

**Fig. 2:**
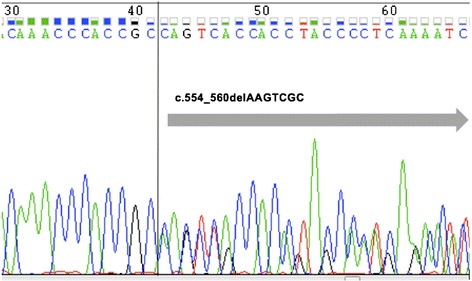
Capillary electropherogram obtained from the ABI PRISM 3700 genetic analyzer. Double peaks are observed from the nucleotide at marker position 42 (indicated above), revealing 2 different sequences: Sequence #1: AA GTC GCC ACC TAC CAC AGA GCC AT (wild-type). Sequence #2: -- --- --C ACC TAC CAC AGA GCC ATC AAA ATC (7-bp deletion)

A 27-year-old Malay woman presented at our hematology clinic with a fever and sore throat. She had a history of hereditary thrombocytopenia that affected her father and 3 brothers. Preliminary clinical investigation at diagnosis revealed abnormal levels of hematological markers - the patient had a white blood cell count of 1.97 × 10^9^ /L (normal: 3.4–9.6 × 10^9^ /L), hemoglobin levels of 7.0 g/dL (normal: 10.9–15.1 g/dL) and platelet count of 20 × 10^9^ /L (normal: 132–372 × 10^9^/L). On morphological analysis, her bone marrow was moderately cellular, with dysplastic changes as well as increased numbers of myeloblasts and monoblasts/promonocytes, indicative of AML with myelodysplastic changes (Fig. 1a). Initial molecular testing by conventional Sanger sequencing revealed the same novel *RUNX1* mutation in the patient, her father and 3 brothers (Fig. 2). A buccal analysis also confirmed that the *RUNX1* mutation identified in this FPD/AML patient is germline in nature.

The patient received standard induction chemotherapy that consisted of high-dose anthracycline and cytarabine in a 3 + 7 regimen and subsequently attained complete morphological remission. Post-induction evaluation of her bone marrow revealed a regenerating bone marrow in morphological remission with myelodysplastic changes (Fig. 1b), as well as 0.3% residual disease, measured using Flow Cytometry. Thereafter, she was given consolidation chemotherapy with cytarabine. As no matched sibling donor or matched unrelated donor was available, she received a haploidentical stem cell transplant (haplo-SCT) from her mother, who is *RUNX1*-mutation negative. While she engrafted on Day 15 of her transplant, her admission was complicated by *Klebsiella* bacteraemia, acute Graft-versus-Host Disease (GvHD) of her liver and Cytomegalovirus reactivation. Post-haplo-SCT, she was given Tacrolimus, Mycophenolate Mofetil and Methotrexate as GvHD prophylaxis. A post-transplant bone marrow aspirate after count recovery showed a normocellular reactive marrow (Fig. 1c) and 0% residual disease on Flow Cytometry.

**Fig. 3:**
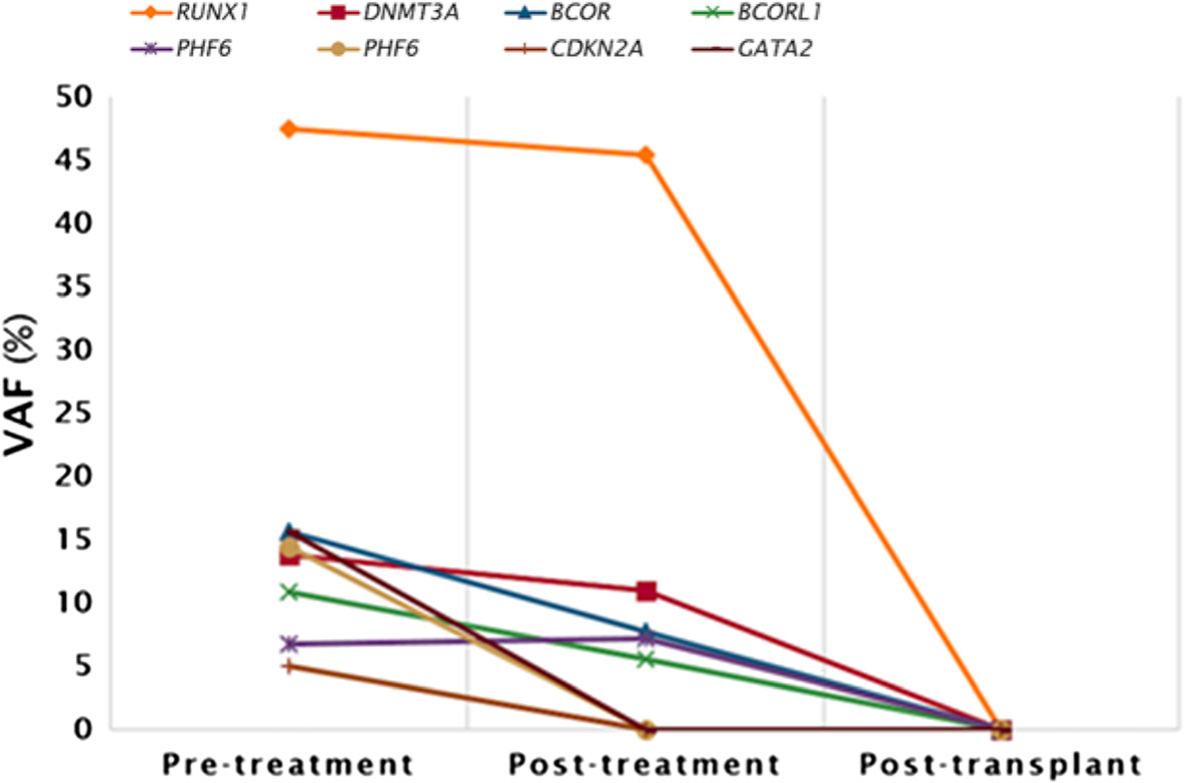
Mutations detected in pre-treatment, post-treatment and post-transplant PB or BM samples

With informed consent obtained from the patient, a comprehensive genomic mutational analysis on a targeted next-generation sequencing (NGS) platform was conducted using either peripheral blood (PB) or bone marrow (BM) samples collected at three different stages – at diagnosis (pre-treatment), post-treatment (after induction and consolidation chemotherapy) and post-haplo-SCT. These time points coincided with the bone marrow examinations done for the patient, as mentioned above. In brief, 568 amplicons (‘tiles’) covering 54 genes that were previously implicated in myeloid neoplasms were assessed for the presence of genomic variants. At each stage, the patient’s genomic DNA was extracted and processed using the TruSeq Custom Amplicon (TSCA) assay for the TruSight Myeloid Sequencing Panel, according to a previously described methodology [12, 13]. The 54 genes evaluated in this targeted gene panel were as follows: *ABL1*, *ASXL1*, *ATRX*, *BCOR*, *BCORL1*, *BRAF*, *CALR*, *CBL*, *CBLB*, *CBLC*, *CDKN2A*, *CEBPA*, *CSF3R*, *CUX1*, *DNMT3A*, *ETV6*, *EZH2*, *FBXW7*, *FLT3*, *GATA1*, *GATA2*, *GNAS*, *HRAS*, *IDH1*, *IDH2*, *IKZF1*, *JAK2*, *JAK3*, *KDM6A*, *KIT*, *KMT2A*, *KRAS*, *MPL*, *MYD88*, *NOTCH1*, *NPM1*, *NRAS*, *PDGFRA*, *PHF6*, *PTEN*, *PTPN11*, *RAD21*, *RUNX1*, *SETBP1*, *SF3B1*, *SMC1A*, *SMC3*, *SRSF2*, *STAG2*, *TET2*, *TP53*, *U2AF1*, *WT1* and *ZRSR2*. After sequence alignment against the reference GRCh37/hg19 human genome assembly, BAM and VCF files were produced by the TruSeq Amplicon software (V.1.1.0.0). Genome annotation was done on the VCF files using the Illumina VariantStudio (V.2.2). Only variants that met the following criteria were selected: non-synonymous mutation, variant not present in dbSNP, read-depth > 100×, variant-allele frequency (VAF) > 5% and acceptable sequence quality. The comprehensive list of mutations, including the hitherto unreported germline *RUNX1* mutation, identified in the pre-treatment, post-treatment and post-haplo-SCT blood samples can be found in (Table 2; Fig. 3). A total of 8 mutations were detected in the pre-treatment sample when she had AML, 5 mutations remained on her post-chemotherapy analysis where there was residual myelodysplasia, and no mutations were present after haplo-SCT. At the time of writing, she is currently 14 months post-transplant, with no evidence of disease relapse, with 100% Donor chimerism.

**Table 2:** Variants identified in the patient pre-treatment, post-treatment and post-transplant

	Gene	Chromosome	Genomic Coordinate	Variant (Genomic)	Variant Type	VAF pre-treatment (%)	VAF post-treatment (%)	VAF post-transplant (%)
1	*RUNX1*	21	36,231,823	NM_001754.4:c.554_560delAAGTCGC	Deletion	47.5	45.4	0
2	*GATA2*	3	128,200,112	NM_032638.4:c.1193G > A	Single-nucleotide substitution	15.6	0	0
3	*DNMT3A*	2	25,463,182	NM_022552.4:c.2311C > T	Single-nucleotide substitution	13.9	11.0	0
4	*BCORL1*	X	129,147,461	NM_021946.4:c.713_714insA	Insertion	10.9	5.6	0
5	*BCOR*	X	39,931,609	NM_001123385.1:c.2989delG	Deletion	15.7	7.7	0
6	*PHF6*	X	133,547,693	NM_032458.2:c.585 + 7delA	Deletion	6.7	7.2	0
7	*PHF6*	X	133,551,319	NM_032458.2:c.955C > T	Single-nucleotide substitution	14.5	0	0
8	*CDKN2A*	9	21,971,137	NM_001195132.1:c.221A > C	Single-nucleotide substitution	5.1	0	0

## Discussion and conclusions

Germline *RUNX1*-mutated FPD/AML, albeit rare, is a relatively well-established entity in myeloid neoplasms. The key publications on novel germline *RUNX1* mutations identified in different FPD/AML pedigrees are listed in Table 1. However, the understanding of the molecular evolution in clonal hematopoietic cells of germline *RUNX1*-mutated patients that led to AML development remains unclear. It is known that less than half of the germline *RUNX1*-mutated patients with hereditary thrombocytopenia eventually develop AML [11] and for those FPD cases that do transform into myelodysplastic syndrome/AML (MDS/AML), the latency period can range from 6 to 76 years [14]. This is a strong indication that second-hit mutational events, on top of the primary germline *RUNX1* mutation, are required for leukemogenesis to occur. In light of this, we herein report potential preleukemic and second-hit mutational events in an FPD/AML patient who was detected with a novel 7-bp germline *RUNX1* deletion.

Firstly, to the best of our knowledge, we identified a hithertho unreported 7-bp germline *RUNX1* deletion (c.554_560delAAGTCGC) in this FPD/AML patient that has not been previously documented in ClinVar (http://www.clinicalgenome.org/data-sharing/clinvar/). Given that the same *RUNX1* deletion was also detected in the patient’s father and 3 brothers, we essentially identified a novel germline *RUNX1* mutation in another FPD/AML pedigree. The patient’s father and 3 brothers all have thrombocytopenia with no evidence of MDS or AML, and are currently on follow-up at our clinic to monitor for onset of myeloid malignancies. This germline *RUNX1* mutation is most likely monoallelic, as the VAF is close to 50%. This is in accordance to previous reports of heterozygous germline *RUNX1* mutations in FPL/AML pedigrees that indicated *RUNX1* haploinsufficiency [6–8, 15].

It is known that *RUNX1*, also known as *CBFα2* or *AML1*, is a master transcription factor that plays a crucial role in regulating the expression of several genes involved in hematopoiesis [16, 17], such as *GM-CSF* [18] and *c-Mpl* [19]. In fact, *RUNX1* is found to be required in the initiation of definitive hematopoiesis during embryological development [16], where the knockout of *RUNX1* gene in mice led to a complete lack of liver hematopoiesis, culminating in CNS hemorrhage and death typically at embryonic day 12.5 [20]. Physiologically, it is most probable that a typical loss-of-function heterozygous *RUNX1* mutation such as the one identified in this patient would, at the very least, disrupt hematopoietic differentiation [21], as with other Class II mutations [2, 22].

The 7-bp germline frameshift mutation identified in our patient affects the Runt domain of the *RUNX1* protein. Most previously reported germline *RUNX1* mutations in FPD/AML also implicate the Runt domain [6–8, 10, 23, 24]. In brief, the *RUNX1* protein constitutes the *α*-subunit of the core binding factor (CBF) [25] and the Runt domain is a DNA-binding domain that binds to a TGT/cGGT consensus sequence in the promoter or enhancer region of the hematopoietic target genes that *RUNX1* regulates [23]. Notably, the Runt domain also binds to the non-DNA binding *β*-subunit of CBF (CBF*β*) to form a crucial heterodimeric transcriptional complex that significantly enhances *RUNX1* DNA-binding affinity [25–28]. The 7-bp frameshift *RUNX1* deletion identified in our patient results in a truncated protein that lacks the C-terminal moiety of the Runt domain and is therefore likely to be a loss-of-function mutation. Given *RUNX1* haploinsufficiency [6], we believe that this would probably result in insufficient levels of *RUNX1* protein product synthesized for proper hematopoietic regulation. As such, this can explain our patient’s history of congenital thrombocytopenia.

On targeted NGS analysis of the patient’s molecular profile, we identified a total of 8 mutations, including the germline 7-bp *RUNX1* deletion, in the pre-treatment sample, of which 5 were found in the post-treatment sample and none were detected in the post-haplo-SCT sample (Table 2, Fig. 3). The 8 mutations detected during the pre-treatment molecular analysis included *RUNX1* (germline), *GATA2*, *DNMT3A*, *BCORL1*, *BCOR*, 2 *PHF6* and *CDKN2A* mutations. The 5 mutations that persisted in post-treatment molecular analysis were *RUNX1* (germline), *DNMT3A*, *BCORL1*, *BCOR* and 1 out of 2 *PHF6* mutations and the 3 mutations that were absent in the post-treatment sample were *GATA2*, *CDKN2A* mutations and a separate *PHF6* mutation. The 3 mutations that were eradicated with induction chemotherapy were most probably somatic leukemic driver mutations that were only found in leukemic cells. Therefore, when complete morphological remission was attained, these 3 driver mutations were lost along with the malignant cells. On the contrary, the 5 mutations which were still present post-treatment were likely to be preleukemic mutations that are typically resistant to induction chemotherapy [11, 29]. The preleukemic mutant cells also seem to retain the ability to differentiate into mature cells [11].

In brief, a preleukemic clone can be described as a subpopulation of hematopoietic progenitor cells with a distinct aberrant molecular profile, which predispose individuals with it to AML development. The mutations carried in the preleukemic clone are not sufficient to cause AML and acquisition of secondary mutational events would be necessary. In our patient, it seems that the preleukemic clone contained *RUNX1* (germline), *DNMT3A*, *BCORL1*, *BCOR* and *PHF6* mutations and the secondary mutations acquired that drove AML transformation were likely *GATA2*, *CDKN2A* and an additional *PHF6* mutation.

While the presence of chemotherapy-resistant leukemic driver mutations is possible, germline *RUNX1* mutations [11] and *DNMT3A* mutations are well-reported preleukemic events [29, 30]. In fact, *DNMT3A* mutations have also been found to persist after complete morphological remission in AML [31, 32]. But to the best of our knowledge, *BCORL1*, *BCOR* and *PHF6* mutations have not been reported as preleukemic mutations in FPD/AML and require further characterization. It was previously reported that patients with persisting mutations at complete morphological remission have a less favorable event-free and overall survival, as compared to patients without such mutations [33]. However, *DNMT3A* mutations detected at complete remission have not been found to affect survival outcome [31, 34]. Therefore, further studies would need to be conducted to better define mutations found in preleukemic clones and more importantly, determine the prognostic significance of such mutations. This may then provide a basis to screen AML patients for specific preleukemic mutations post-induction chemotherapy to guide further treatment decision.

Interestingly, in 2014, a Japanese study identified somatic *CDC25C* and *GATA2* mutations as sequential preleukemic and secondary mutational events respectively, in the malignant transformation of germline *RUNX1*-mutated FPD/AML [35]. However, *CDC25C* mutations were not detected in a subsequent study in the United States, possibly due to ethnic differences [36].

In our patient, we identified *GATA2*, *CDKN2A* and *PHF6* mutations as potential secondary leukemic driver mutations in germline *RUNX1*-mutated AML. Of note, *PHF6* mutations are known to be closely associated with *RUNX1* in myeloid neoplasms but in a different clonal evolution setting where *PHF6* mutations precede *RUNX1* mutations [37]. In general, second-hit mutational events responsible for propelling a preleukemic clone in germline *RUNX1*-mutated FPD/AML into frank leukemia are not commonly reported. Preudhomme et al. (2009) identified secondary *RUNX1* somatic mutations as potential second-hit events in germline *RUNX1*-mutated FPD/AML [24] and subsequently, Shiba et al. (2012) reported an acquired *CBL* mutation as a possible second-hit mutational event in a case of FPD transformation to Chronic Myelomonocytic Leukemia (CMML) [38].

Overall, there were no concomitant driver mutations in de novo AML such as *NPM1* or *FLT3* mutations detected in our germline *RUNX1*-mutated FPD/AML patient, in accordance to what was previously reported [11]. More importantly, the Class II germline *RUNX1* mutation in this patient was not accompanied by a Class I mutation. This suggests that an alternative to the conventional “2-hit” model for AML pathogenesis [2] that requires the presence of both Class I and Class II mutations is required in FPD/AML cases.

In summary, we identified a novel 7-bp germline *RUNX1* deletion in an FPD/AML patient that is found in the important Runt domain of *RUNX1*. Through molecular analysis, we also identified possible preleukemic and second-hit mutational events implicated in the pathogenesis of germline *RUNX1*-mutated AML. Further studies at the cohort level are required to assess the prevalence of these putative preleukemic and secondary mutations in FPD/AML. In addition, prognostic significance of preleukemic mutations that persist after complete remission needs to be established. With better classification of mutations implicated in FPD/AML and other types of AML into preleukemic and second-hit events, this can potentially improve molecular risk stratification of AML patients that can guide disease management.

## Abbreviations

AML: Acute Myeloid Leukemia
BM: Bone marrow
CBF: Core binding factor
FPD/AML: Familial Platelet Disorder with predisposition to Acute Myeloid Leukemia
GvHD: Graft-versus-host Disease
Haplo-SCT: Haploidentical stem cell transplant
MDS/AML: Myelodysplastic Syndrome/Acute Myeloid Leukemia
NGS: Next-generation sequencing
PB: Peripheral blood
TSCA: TruSeq Custom Amplicon
VAF: Variant-allele Frequency
